# Importance of frequency and intensity of strength training for work ability among physical therapists

**DOI:** 10.1038/s41598-022-18539-4

**Published:** 2022-09-02

**Authors:** Joaquín Calatayud, Álvaro Morera, Yasmín Ezzatvar, Rubén López-Bueno, Lars L. Andersen, Ferran Cuenca-Martínez, Luis Suso-Martí, Enrique Sanchís-Sánchez, Laura López-Bueno, José Casaña

**Affiliations:** 1 Exercise Intervention for Health Research Group (EXINH-RG), Department of Physiotherapy, University of Valencia, C/ Gascó Oliag 5, 46010 Valencia, Spain; 2 National Research Centre for the Working Environment, Copenhagen, Denmark; 3 Department of Physical Medicine and Nursing, University of Zaragoza, Zaragoza, Spain; 4 Sport Sciences, Department of Health Science and Technology, Aalborg University, Aalborg, Denmark

**Keywords:** Health occupations, Risk factors

## Abstract

The aim of the study was to evaluate the association between frequency and intensity of strength training participation and work ability among physical therapists (PTs). The Work Ability Index questionnaire (WAI) and a questionnaire about participation in strength training during leisure time were administered to a sample of Spanish PTs. In addition, participants provided information on gender, age, body mass index, education, substance use, working experience and working hours per week. The odds for having excellent WAI (score 44–49) as a function of intensity or frequency of strength training participation were determined using binary logistic regression controlled for various confounders. Data from 981 PTs were analysed. High-intensity strength training (> 80% 1 RM) showed strong associations with excellent WAI (odds ratio = 9.7; 95% confidence interval, 2.9–31.6). In addition, performing strength training more than 3 times per week was associated with excellent WAI (odds ratio = 1.79; 95% confidence interval, 1.24–2.59), however, no significant associations were found with lower levels of frequency and intensities. High-intensity strength training 3 times per week is associated with excellent WAI among PTs. Training programs meeting these features may importantly contribute to maintain or improve WAI.

## Introduction

Health-care professionals are an important part of the total workforce, and physical therapists (PTs) are an essential part of them. Currently in Europe, there are more than half a million PTs working, representing more than 8% of the total health-care workforce^1^, with Spain being one of the European countries with more practising PTs per 100,000 inhabitants during the last years^1^.

According to previous studies, high prevalence of suffering musculoskeletal disorders (MD) have been registered in health-care professionals as a result of the physically demanding nature of their tasks^2^. These MD are considered one of the most significant causes of disability, early retirement^3^, increased health care use^4^, reduced work productivity, lower levels of health-related quality of life^5^ and are a significant threat to work ability^6^. In fact, a recent cohort study added that a poor work ability (defined as the balance between the individual physical and mental capacity, and the job demands) increased the risk of rehabilitation, using unemployment benefits, having fewer employment days and less income from regular employment, and also increased risk of premature death^7^.

A previous study compared the risk of MD among 10 groups of medical personnel in Taiwan and showed that PTs, and nurses had the highest risk for work-related MD^8^. Actually, as demonstrated by a systematic review, work-related MD affect at least 50% and up to 90% of PTs during their careers^2^. In addition, a recent observational study among PTs found a strong association between pain intensity and levels of work ability^9^, which may lead to premature exit from the labour market^10^, long term sickness absence and disability pension^11^.

All these findings may be related to the physical exigencies of PTs while working and the exposition to multiple factors like sustained awkward positions, dealing with dependent patients, repetitive tasks, high force manual techniques for treating patients, techniques that exert direct pressure on certain joints during the treatment, high mental demands and stress^2,12^. However, modifiable individual lifestyle factors such as physical activity may impact on work-related MD and, consequently, in work ability. For instance, the use of physical exercise, and especially strength training seems promising as proper options to improve work ability, as well as to prevent the deterioration of health and physical capacity among workers with MD, and with a physically demanding work^13^. However, literature is scarce regarding factors that may lead to have excellent work ability. In fact, no previous studies have evaluated the relation between frequency and intensity of self-reported strength training participation for having excellent work ability, neither among PTs nor among other workforces. This information could be useful to design effective strategies and interventions to improve work ability among PTs or prevent its deterioration.

The aim of the present study was to evaluate the association between frequency and intensity of self-reported strength training participation and work ability among PTs. It was hypothesized that high-intensity strength training rather than low-intensity or strength training frequency would be associated with better work ability.

## Methods

This cross-sectional study was carried out in 2017, as part of a research evaluating the working environment among PTs. Registered PTs from different professional associations from Spain were invited to participate. We excluded PTs that were already retired or were not actively working. The study conformed to the Declaration of Helsinki and was approved by the local ethics committee of the University of Valencia. Informed consent was obtained from all subjects and all data of the study were treated anonymously. This article adheres to the Strengthening the Reporting of Observational studies in Epidemiology guidelines (STROBE).

### Procedures

We sent an e-mail to the members of different professional PT associations in Spain, inviting them to voluntarily participate in the study. The e-mail explained the purpose of the study and included a link to complete the questionnaire. After a month, we sent a reminder e-mail inviting PTs to participate if they had not done already. Due to the recruiting procedure (since we sent the questionnaire to the different associations but only analysed the responses), the exact number of invited participants was unknown. By responding to the questionnaire, participants gave consent to participate in the study and permission for the results to be published. The name and correspondence of the researchers was included in the cover letter for solving any doubt or concern.

### Questionnaire content

Before sending the definitive questionnaire, this was pilot tested by 10 PTs from different settings, who reviewed each question and provided feedback. Once the questions were revised and modified when necessary, an online questionnaire was prepared by using “Google Forms” (Google Inc, Mountain View, CA) for the response compilation and the data storage. Due to data privacy reasons, the setting of the survey system was set to “anonymous”; that is, it was not possible to link the individual responses to the individual e-mails of the participants. From the questionnaire, information on gender, age, body mass index, education, and substance use were extracted.

### Work ability Index

Work ability was measured using the Work Ability Index questionnaire, which includes the following subscales: (1) Current work ability in comparison to lifetime best, (2) work ability in relation to the physical and mental demands of the job, (3) number of current diseases diagnosed by a physician, (4) estimated work impairment due to diseases, (5) sick leaving during the past year, (6) own prognosis of work ability two years from now, and (7) mental resources. The final score was calculated by summing up the estimated points for each item^14^. Work Ability Index score ranges from 7 to 49 points, determining four different categories: poor work ability (7–27 points), moderate work ability (28–36), good work ability (37–43) and excellent work ability (44–49 points). The internal validity of this questionnaire has been previously evaluated, with a proper relationship between its subjective results and more objective assessments^15^, as well as a satisfactory test–retest reliability^16^.

### Levels of strength training during leisure time

Participants were asked about their involvement in strength training by using the following questions: “During a typical week, do you do any physical activity at your leisure time specifically designed to strengthen your muscles, such as weightlifting, elastic-band training, push-ups... ?” Those who answered “yes” were also asked about training frequency and intensity. We defined frequency as the number of training sessions/week and was categorized as 0, 1 to 2, or ≥ 3. Intensity was defined as the magnitude of the effort during a typical training session (i.e., intensity predominating in your training program), based on their RM, with 3 possible answers: ≤ 50% 1RM, 51% to 79% 1RM, or ≥ 80% 1RM. All these cut points were established according to general strength training guidelines^17,18^.

### Statistical analysis

The data analysis for this paper was generated using SAS software (SAS Institute Inc, Cary, NC), Version 9.4 of the SAS System for Windows. Copyright © 2021 SAS Institute Inc. SAS and all other SAS Institute Inc. product or service names are registered trademarks or trademarks of SAS Institute Inc., Cary, NC, USA. Descriptive statistics were used to report demographic characteristics of the participants, including age, body mass index, gender, education, smoking, alcohol units per week, and work ability. Using binary logistic regression, odds ratios (ORs) and 95% confidence intervals were calculated for having excellent work ability (score 44–49) as function of frequency (0, 1–2, and ≥ 3 times/wk, respectively) and intensity (≤ 50% 1RM, 51%–79% 1RM, and ≥ 80% 1RM, respectively) of strength training as mutually adjusted independent variables (reference category: 0 min/wk for the frequency and ≤ 50% for the intensity), while adjusting for confounding factors (age, gender, education, work experience, and working hours per week). Analyses concerning intensity were restricted to those participants with data on such outcome.

According to a previous study that compared ORs with effect sizes (Cohen d), ORs of 1.68, 3.47, and 6.71 correspond to small, medium, and large effect sizes, respectively^19^. Because we evaluated effects rather than associations, we decided to use the terms “weak,” “moderate,” and “strong” positive associations for ORs of 1.68, 3.47, and 6.71, respectively. For ORs of < 1, the reciprocal of the OR should be considered; that is, ORs of 0.60, 0.29, and 0.15 correspond to weak, moderate, and strong negative associations, respectively.

## Results

A total of 1006 responses were obtained, but 25 questionnaires had to be excluded from analysis due to missing data in at least one of the main outcomes. As a consequence, we analysed data from 981 questionnaires. Most of the participants were women (70.6%) and the mean age of the study population was 34.3 ± 8.0 (SD) years. The average score of the work ability questionnaire was 41 ± 4.9 (SD) points, with 32.7% of the participants having an excellent score. Table 1 shows demographic data of the participants.

**Table 1:** Demographic data (N = 981).

	N	Mean	SD	%
**Gender**				
Men	288			29.4
Women	693			70.6
**Highest education level**				
Bachelor (3-year)	479			48.8
Bachelor (4-year)	236			24.1
Master	258			26.3
PhD	8			0.8
**Smoking**				
No	852			86.9
Yes	129			13.2
Age (years)	981	34.3	8.0	
BMI (kg m^−2^)	981	23.3	3.4	
Alcohol (units per week)	981	2.2	2.3	
Work ability	981	41.0	4.9	
**Work ability**				
Poor	30			3.1
Moderate	190			19.4
Good	440			44.8
Excellent	321			32.7
**Working experience**				
0–5 years	251			25.6
6–15 years	480			48.9
> 15 years	250			25.5
**Working hours per week**				
< 35	310			31.6
35–45	555			56.6
> 45	116			11.8

The ORs for having excellent work ability (in comparison of having poor, moderate and good work ability as a referent) regarding the frequency and intensity of strength training participation are detailed in Table 2.

**Table 2:** ORs for having excellent work ability (analyses controlled for gender, education, experience, and working hours per week) (reference: no excellent workability).

	N	%	Excellent work ability
			OR (95% CI)
**Strength training frequency**			
0·wk^−1^	600	61.2	1
1–2·wk^−1^	206	21.0	1.30 (0.90–1.86)
> = 3·wk^−1^	175	17.8	**1.79 (1.24–2.59)**
**Strength training intensity**			
< 50%	143	36.7	1
60–70%	220	56.4	1.40 (0.83–2.38)
> 80%	27	6.9	**9.71 (2.98–31.62)**

Bold letters denote statistically significant result.

In relation with the frequency of the strength training, with no strength training per week as a reference, a positive weak association for having excellent work ability levels was found in those PTs who reported performing more than 3 strength training per week. However, the odds for having excellent work ability were not significantly higher among PTs who trained 1–2 days per week.

For the intensity of the training, the strongest association for having excellent work ability was showed in those PTs who executed high-intensity training (> 80% 1 RM). In contrast, performing lower intensities (< 50% 1RM and 60–70% 1 RM), was not significantly associated with excellent work ability levels.

## Discussion

The main findings of the present study are that performing high-intensity strength training (> 80% 1RM) is strongly associated with having excellent work ability levels among PTs. Training more than 3 times per week was also significantly associated with excellent levels of work ability, although these associations were weaker. No significant associations were found between excellent work ability and lower levels of frequency and intensities.

As hypothesized, performing high-intensity strength training (> 80% 1RM) during leisure-time is strongly associated with excellent levels of work ability among PTs. In accordance with our results, a cross-sectional study among 3000 workers with physically demanding jobs concluded that the duration of high-intensity physical activity during leisure time was positively associated with work ability^20^. In addition, authors found that those who performed ≥ 5 h of high-intensity physical activity per week had on average 8 points higher work ability than those who did not perform such activities. However, since the aforementioned study assessed general physical activity, it is unknown whether strength training could specifically influence their results.

In line with our findings, previous experimental literature seems to be consistent with the positive effect of high-intensity strength training in preventing work ability decrease. For instance, a study revealed that performing brief sessions of high-intensity strength training three times a week at the workplace during 10 weeks prevented a further decline in work ability among slaughterhouse workers with chronic musculoskeletal pain^21^. Furthermore, another study^22^ showed how an intensive strength training program was associated with increased self-rated work ability and improved mental health among female workers on long term sick leave. However, the intensity used during the intervention was not reported^22^.

Interestingly, our analysis did not reveal any association between a low-to-moderate intensity strength training participation with having excellent work ability. These results are in accordance with several studies. For instance, a randomized controlled trial among construction workers did not reveal any significant improvement in work ability after a 12-week exercise intervention of aerobic capacity training (at least 70% of Vo2max) and moderate-strength training (60% 1RM) 1 h a week^23^. Moreover, other authors found no significant improvements in work ability after 8-month moderate worksite exercise that involved muscle strengthening, cardiovascular exercise, and stretching once a week among laundry workers^13^. However, their mean work ability score was high (40 out of 49 points) among the participants, and this may explain the absence of difference between groups^13^. In addition, our results are also in agreement with a systematic review which observed that the lack of leisure-time vigorous physical activity is an important factor associated with a poor work ability^24^.

Nonetheless there is not a clear mechanism that could explain our results. One hypothesis could be related with the elevated risk of developing MD among health-care professionals due to the physically exhausting essence of their work job^2^. It has been demonstrated that musculoskeletal pain is what leads to a lower work ability and not vice versa^6,25^. For these reasons and since high-strength training is strongly associated with lower levels of musculoskeletal pain^14^, its reduction could explain our results. Likewise, those PTs who performed high-intensity strength training during their leisure time may be better prepared to confront the demanding physical tasks of their profession and reduce, in consequence, work-related disorders and therefore experience a better work ability. Another point could be related to the fatigue and the physical exposures during their work tasks. It is possible that those PTs with more frequently and more intense training usually have more leisure-time to do it. In addition, those who work harder or longer during working time might not have the time or the energy to train hard and have an effect on strength training. In fact, one study has reported how being exposed to diverse physical exposures during work increases bodily fatigue^26^. Besides, another article showed a relation between being more than a quarter of the workday exposed to many physical work demands and lower work ability^27^.

In relation to the frequency of strength training participation, our results have reported a weak relation between an excellent work ability and those PTs who performed more than 3 strength training per week. Conversely, no association was found in those who trained 1–2 days per week. Some studies support that muscle mass and strength adaptations can be attained despite different training frequencies of the strength training program when total training volume is equated in healthy males and females beginning a strength training program^28,29^. In line with our findings, the previous studies seem to be consistent with performing a strength training program more than 3 times a week and its effectiveness on having better levels of work ability. For instance, some authors showed signs of work ability improvement among workers with chronic pain and work disability after implementing high-intensity strength training 3 or more times a week^22,30^. Conversely, authors could not expose any significant improvement in work ability after a 3 days × 20 min exercise intervention during 12 weeks^23^. This might be attributed to the characteristics of the intervention, which was mainly focused on aerobic training and the strength training time was reduced. Interestingly, it was reported that a change in aerobic capacity may have only a limited effect on work ability^23^. This could be explained since physically demanding tasks in many jobs (e.g., PTs) do not usually require a high cardiorespiratory capacity.

Regarding the strength training less than 3 times per week, only a study conducted among female workers with no chronic pain or disability could follow the results we have obtained. In this article the authors failed to see a change in work ability after performing 1 year of moderate guided worksite exercise (strength training, aerobic training and stretching) once a week among female laundry workers. However, the authors concluded that perceived work ability cannot be affected very positively using a single component exercise intervention and argued that work ability promotion may need a more multifactorial approach and that the high score levels of work ability at baseline obtained by the participants (40 out of 49) may explain the results of no differences between the groups^13^. On the contrary, another study have showed the positive effects of executing supervised exercise twice a week for preventing a further decline in work ability among workers with chronic musculoskeletal pain and among women performing physically demanding home care work^28^.

The present study has some limitations and strengths. The cross-sectional nature of this study is the principal limitation due to the exposure and outcomes were concurrently assessed and it cannot determine causality. Furthermore, as younger participants spend more time online, they may have been more predisposed to participate than their older counterparts, considering that the questionnaire was online. Another limitation may be the avoidance of PTs with low work ability due pain-related health problems to use high-intensity strength training because of the pain. Hence, caution should be taken when extrapolating the results to other populations with a different distribution of work ability values. We did not account for training volume and thus future studies should explore its influence on work ability. It is plausible that the intensity results are more inaccurate for those with less training experience or who do not test their 1RM or do not know how to translate a certain number of repetitions into percentages of 1RM. In addition, since the survey forced participants to select a single intensity, when in fact many trainees may use multiple repetition zones in a typical training week, it is plausible that their answers does not reflect their training intensity accurately. However, all the cut points for intensity zones were established according to general strength training guidelines^17,18^. Importantly, this is an efficient way of assessing strength training participation to multiple participants, since for a self-reported questionnaire, it would be difficult to explain a perfectly detailed training dosing that covers their leisure time (as occurs with general physical activity questionnaires). There are a variety of cultural differences among countries and regions such that these results may not be replicated elsewhere. However, this is the first study to measure work ability in a large sample of PTs, thus no reference values exist to compare with. In this sense, we can compare the results to another large-scale study^31^ that found 41% of the population to have excellent work ability, a higher value than the one we found. An additional significant limitation was the utilization of a self-reported measure, so the results could have been underestimated or overestimated by the social desirability or overcall bias. Moreover, selection bias could lead to an overrepresentation of some groups, especially those who exercise more than once a day, as a result of reporting frequency as the number of training days per week, which might lead to an inaccurate association estimate. In this sense, future studies should consider asking about average training time in a session. It could also be interesting avoiding to stablish a training frequency cut point of 3 days or more, so a more specific frequency can be captured. A strength of our study was the large sample size. However, since the exact number of invited participants was unknown, we have not been able to provide the response percentage. Importantly, our study enhances the current understanding of the importance and relevance of practicing high-intensity strength training for PTs. According to a recent survey, most of the PTs frequently prescribe strength training in spite of low knowledge scores^32^. Hence, increase their knowledge on strength training could positively impact their own participation, which ensures greater emphasis in professional and continuing education^32^.

In conclusion, our findings suggest that PTs should engage high-intensity strength training (≥ 80% 1RM) during leisure time to maintain excellent levels of work ability. In addition, training 3 or more days per week could have a small positive effect too.

## Data Availability

Data are available from the authors upon reasonable request (contact: joaquin.calatayud@uv.es).

## Abbreviations

PTs: Physical therapists
MD: Musculoskeletal disorders
